# Investigating a Potential Link Between Metabolic-Neurotrophic Coupling in Breast Tumor Innervation in Bioengineered Models

**DOI:** 10.3390/cells15211959

**Published:** 2026-10-28

**Authors:** Nikolas Ala-Kokko, Emory Gregory, Alan Woessner, Bing Guan, Winifred Middleton, Donald J. Johann, Narasimhan Rajaram, Timothy J. Muldoon, Younghye Song

**Affiliations:** 1 Department of Biomedical Engineering, University of Arkansas, Fayetteville, AR 72701, USA; 2 Arkansas Integrative Metabolic Research Center, University of Arkansas, Fayetteville, AR 72701, USA; 3 Winthrop P Rockefeller Cancer Institute, University of Arkansas for Medical Sciences, Little Rock, AR 72205, USA

**Keywords:** breast cancer, tumor innervation, metabolism, neurotrophin, metabolic-neurotrophic coupling, tumor microenvironment, tissue engineering

## Abstract

**Highlights:**

**What are the main findings?**

- Human breast cancer cells across different stages exhibit unique neurotrophin and cognate receptor expression profiles in a stromal cell presence and metabolism-dependent manner.
- Breast tumor innervation may be heightened early in the pre-malignant stage, in part with assistance from stromal cells in the tumor microenvironment.

**What are the implications of the main findings?**

- Stage-specific metabolic-neurotrophic coupling may contribute to breast tumor innervation and warrants further, in-depth investigation.
- The impact of stromal cells in aiding tumor innervation needs further attention.

**Abstract:**

Metabolic reprogramming is a well-established hallmark of cancer, contributing to adverse behaviors like invasion, metastasis and recruitment of tissue-resident cells to the tumor microenvironment. Among recruitment capabilities, innervation has emerged as an active contributor of disease progression. Despite association with poor patient prognosis, mechanisms governing nerve recruitment at various stages of progression remain poorly understood. This study investigates how different stages of breast cancer cells are associated with the metabolic link of cancer and their pro-axonogenic capability. Here, we used human isogenic MCF10 mammary epithelial cell line series, three-dimensional collagen hydrogels, human adipose stromal cells (ASCs) and freshly isolated rat sensory neurons. Multiphoton optical redox imaging coupled with ATP production rate assays validated direct metabolic inhibition and highlighted the utilization of glutamine in progressed cells. We identified the expressions of brain-derived neurotrophic factor (BDNF) and glial cell line-derived neurotrophic factor (GDNF) from pre-malignant and malignant cultures, respectively. Interestingly, when treated with metabolic inhibitors, monocultures of MCF10 cells reduced neurotrophin release while cocultures with ASCs showed no or even elevated neurotrophin secretion. Together, these findings raise the possibility that breast tumor innervation is shaped in part by stage-specific metabolic-neurotrophic coupling, presenting a concrete mechanistic question for future investigation.

## 1. Introduction

Cancer is the second leading cause of deaths in the United States with a projected death toll of approximately 600,000 in 2026 [1]. While cancer mortality rates continue to decline with novel technologies, the global health burden of 2.5 million new diagnosed breast cancers makes it the most diagnosed form of cancer worldwide [2]. Environmental, hormonal, lifestyle, and genetic risk factors all contribute to incidence, deeming it necessary for routine screenings for early diagnosis and screening. Despite advancements in diagnostic technologies, patient-specific therapeutic strategies are needed for best prognosis. Therefore, understanding serial evolution of cancer helps target the tumors appropriately.

Breast cancer progression from normal epithelium through pre-malignant, ductal carcinoma in situ, invasive ductal carcinoma, and ultimately metastasized tumors correlates with profound morphological and metabolic reprogramming, shaping the tumor microenvironment (TME) and aiding in tumor growth. Initially, cancer metabolism was primarily associated with the Warburg effect, i.e., tumor preferential reliance on glycolysis despite normoxic environment [3,4]. Now, there is a significant appreciation for more complex metabolic functions, including mitochondrial metabolism dynamics and the dependency of glutamine catabolism, referred to as glutamine addiction [5,6,7]. Through glycolysis, the tricarboxylic acid (TCA) cycle, and fatty acid oxidation, nicotinamide adenine dinucleotide (NAD^+^) is reduced into NADH; NADH then contributes to oxidative phosphorylation (OXPHOS) and subsequent ATP production [8]. Alternatively, flavin adenine dinucleotide (FAD) is synthesized from riboflavin and transported into the mitochondria, where it is reduced to FADH_2_ to support OXPHOS and other pathways of mitochondrial metabolism. Oxidation of NADH and FADH_2_ transfer electrons through complexes I–IV of the electron transport chain while protons are pumped from the matrix into the intermembrane space, generating a proton gradient. This gradient across the inner mitochondrial membrane results in an influx of protons back into the mitochondria via ATP synthase, facilitating ATP production [9]. By switching between glycolysis and OXPHOS, aggressive cancers are considered to possess metabolic plasticity [10,11]. Through this mechanism, cancer cells exposed to normoxic, hypoxic, and/or acidic environments as well as nutrient fluctuations demonstrate enhanced survival, in part by exploiting resident stromal cells [12,13]. Understanding how these metabolic shifts occur on a continuum remains undervalued in achieving mechanistic understanding of how this reprogramming educates tumor niche formation, specifically within the pro-axonogenic landscape of the TME.

One rapidly emerging dimension of the TME is cancer–nerve intercommunication, contributing to cancer initiation and progression [14,15,16]. Tumor innervation, in which neurites infiltrate nearby tumors, has been identified in cancers and enhances tumor development [17,18,19]. Tumor innervation results in increased patient pain and poor prognosis, with surgical interventions risking the viability of peripheral nerves [20]. Studies have shown that neuro-stimulatory signals from the TME including brain-derived neurotrophic factor (BDNF), glial cell line-derived neurotrophic factor (GDNF), nerve growth factor (NGF), and neurotrophin 3 (NT-3) further enhance innervation of breast tumors [18,21]. Yet, the specific effects of metabolic rewiring on creating a pro-axonogenic environment leading to breast tumor innervation remain largely unclear. Although metabolic reprogramming and cancer-nerve crosstalk are individually recognized features of the TME, their interplay remains relatively underexplored.

Using isogenic human mammary epithelial cell line panel: MCF10A (10A, healthy), MCF10AneoT (AneoT, premalignant), MCF10DCIS.com (DCIS, early-stage cancer), and MCF10CA1a (CA1a, malignant), we characterized how transcriptional characteristics and metabolic behaviors influence phenotypes to establish innervating tumor niches [22,23,24,25,26]. These cells were first cultured in 3D collagen gels mimicking tumor microarchitecture with or without adipose stromal cells (ASCs), followed by coculture with dorsal root ganglia (DRG) sensory neurons to measure neurite infiltration. Then, we targeted glutaminase (GLS1) and lactate dehydrogenase A (LDHA) with their pharmacological inhibitors, BPTES and FX-11, respectively. We found that AneoT cultures exhibited enhancement of OXPHOS and glycolysis. While healthy cells remained low in metabolic dependence, DCIS cells relied on glycolysis while CA1a cells exhibited signs of glutamine addiction. Interestingly, AneoT primarily secreted BDNF while CA1a predominantly released GDNF. Further, AneoT cultures induced higher degrees of innervation, indicating that axonogenic priming of the TME may occur early before the onset of full malignancy.

Here, we examine how stage-specific metabolic shifts in breast cancer cells correlate with neurotrophin production and neurite outgrowth, testing alongside pharmacological inhibitors of GLS1 and LDHA, to assess whether perturbing these metabolic states sufficiently alters neurotrophin release. In this current study, we address prominent gaps: classifying metabolic and neurotrophin phenotypes across four isogenic stages of breast cancer progression and the capacity of these stages to support tumor innervation through the direct culturing of neurons alongside breast cancer cells, with the addition of ASC contribution, and indirectly via neurotrophic signaling. Together, these data provide a basis for future mechanistic work on how metabolism contributes to cancer–nerve crosstalk during disease progression.

## 2. Materials and Methods

### 2.1. Cell Culture

Four human breast mammary epithelial cell lines: MCF10A, MCF10AneoT, MCF10DCIS.com, and MCF10Ca1a.cl1 were obtained from ATCC (10A) and Karmanos Cancer Institute (AneoT, DCIS.com and CA1a). All were cultured in DMEM/F12 containing L-glutamine and sodium bicarbonate (Fisher Scientific, Waltham, MA, USA, catalog #11320033) supplemented with 5.26% horse serum (Fisher Scientific, catalog #16050122), 1.05 mM CaCl_2_ (Sigma Aldrich, St. Louis, MO, USA, catalog #449709), 10 mM HEPES (Fisher Scientific, catalog #15630106), 10 μg/mL insulin (Fisher Scientific, catalog #12585014), 20 ng/mL epidermal growth factor (Fisher Scientific, catalog #PHG0311), 0.5 μg/mL hydrocortisone (Sigma Aldrich catalog #H0888), 105 μg cholera toxin (Sigma Aldrich catalog #C8052), and 1% penicillin-streptomycin (P/S; Fisher Scientific, catalog #15140163). Human adipose-derived stem cells (ASC) were purchased from Lonza (Walkersville, MD, USA, catalog #PT-5006) and maintained in DMEM/F12 containing glutamine and sodium bicarbonate, supplemented with 10% fetal bovine serum (FBS; Bio-Techne, Minneapolis, MN, USA, catalog #S11150) and 1% P/S. All cells were cultured at 37 °C with 5% CO_2_. STR authentication of MCF10 cell lines was performed by IDEXX BioAnalytics (Columbia, MO, USA).

### 2.2. Three-Dimensional Cell Culture

Collagen hydrogels (10 mg/mL) were produced following a previously established protocol [27]. Collagen gels were diluted to a working concentration of 5 mg/mL in the following order: 10% (*v*/*v*) Medium 199 (Sigma Aldrich, catalog #M0650), neutralized to pH 7.4 with 1M NaOH/HCl, 1× PBS, and 10% (*v*/*v*) complete media with desired cell concentration. Cells were embedded in the hydrogels at a final density of 0.5 million cells/mL. In coculture platforms, MCF and ASCs were each added at 0.25 million cells/mL. Samples were incubated for 10 min on ice, 10 min at room temperature, and 10 min at 37 °C to polymerize for increased fiber thickness as previously demonstrated [28,29]. Silicone microwells were fabricated from 0.01 inch thick sheets (Specialty Manufacturing, Inc., St. Paul, MN, USA) with an 8 mm biopsy punch. The microwells were first plasma cleaned (Harrick Plasma, Ithaca, NY, USA) followed by treatment with 1% (*v*/*v*) polyethylenimine (PEI, Sigma-Aldrich, catalog #181978) for 10 min, 0.1% (*v*/*v*) glutaraldehyde (Sigma-Aldrich, catalog #G6257) for 30 min, and washed with deionized water twice. Cell-laden hydrogel was added to the microwells to create 200 µm thick gels. Cocultures of MCF cells and ASCs were maintained in 1:1 MCF media to ADSC media (coculture media). Cultures were maintained for 5 days for RNA experiments, 7 days for protein experiments and 10 days in total for DRG neurite outgrowth analyses. Media were replaced every 48 h. FX-11 (Sigma-Aldrich, catalog #427218) and BPTES (Sigma-Aldrich, catalog #5.30030) were reconstituted in DMSO per manufacturer’s recommendations and added to cultures at 10 µM. All treatments were started after 72 h from the culture start date and refreshed every 48 h until samples were harvested. All controls had equal volumes of DMSO.

### 2.3. RNA Isolation and Quality Control

Total RNA was extracted from the cells using Quick-DNA/RNATM Miniprep Plus Kit (Zymo Research, Irvine, CA, USA, catalog #D7003) as described in the manufacturer’s instructions. The final RNA concentration was measured by Qubit™ RNA BR Assay (Thermo Fisher Scientific, Waltham, MA, USA, catalog #Q10210). RNA quality was assessed by Fragment Analyzer System (Agilent, Santa Clara, CA, USA) using RNA kit (15 nt) (Agilent, catalog #5191-6572) [30]. DV200 values representing the percentage of RNA fragments above 200 bp in length were determined as described previously.

### 2.4. RNA-Seq Library Preparation and Sequencing

A measure of 0.1–1 µg of total RNA was used as input to generate sequencing libraries using TrueSeq Stranded mRNA Library Prep Kit (Illumina, San Diego, CA, USA, catalog #20020594) following the manufacturer’s protocol in the reference guide. Briefly, total RNA was denatured and mRNA was purified by binding of poly A RNA to the beads. mRNA was then fragmented and reverse transcribed into cDNA. Double-stranded cDNA libraries were prepared by A-tailing, adaptor ligation and index PCR amplification. The concentration of final libraries was assessed by Qubit™ 1X dsDNA HS Assay (Thermo Fisher Scientific, catalog #Q33231). The quality control of libraries was performed on Fragment Analyzer System (Agilent) using HS NGS Fragment kit (1–6000 bp) (Agilent, catalog #5191-6578), and QuantStudio Real-Time PCR system using KAPA Library Quantification Kit (Kapa Biosystems, Wilmington, MA, USA, catalog #KK4824). Libraries were sequenced either on a NextSeq2000 or NovaSeq 6000 (Illumina) with paired-end mode (read1: 101 cycles, read 2: 101 cycles, i7: 8 cycles, i5: 8 cycles).

### 2.5. RNA-Seq Bioinformatics Methods

The Illumina DRAGEN version 4.4.7 was used for BCL file demultiplexing and RNA alignment. DRAGEN BCL conversion was used to generate FASTQ files. FASTQC version 0.11.9 was used for FASTQC analysis. RNA-seq alignment and quantification was performed by the DRAGEN 4.4.7 pipelines. The amount of sequencing per sample was 25 million reads or greater (≥25M reads/sample), following best practices. Differential expression analysis was performed using DESeq2 (version 1.50.2) in R (version 4.5.2). To account for variations in sequencing depth and library composition, raw integer read counts were normalized internally using the median-of-ratios method. Differential expression testing was conducted by fitting a negative binomial generalized linear model to the normalized count data. To control the false discovery rate (FDR) arising from simultaneous genomic testing, raw *p*-values were adjusted using the Benjamini–Hochberg (BH) procedure. Prior to correction, independent filtering was automatically applied to eliminate genes with low mean normalized counts, thereby optimizing statistical power. Transcripts were considered significantly differentially expressed if they met a threshold of an adjusted *p*-value (padj) < 0.05. No filtering threshold was applied to the log2 fold change.

### 2.6. qRT-PCR

Cells were initially liberated from collagen gels via 0.1% collagenase A incubation at 37 °C for 20 min. After three PBS washes, total RNA was isolated from cells using Monarch Total RNA Isolation Kit (New England Biolabs, Ipswich, MA, USA, catalog #T2110L) following the manufacturers’ instructions. A measure of 0.5 µg RNA was reverse transcribed into cDNA. Standard curves analysis was performed on all primers to ensure product viability. All primers were designed and validated prior to experiments were performed (Table 1). Expression of genes was normalized to a housekeeping gene RPLP0.

### 2.7. Multiphoton Microscopy

Optical redox imaging of NADH and FAD autofluorescence was performed using a multiphoton microscope (Bruker Investigator; Bruker, Billerica, MA, USA) with a femtosecond tunable pulsed laser (MaiTai; Spectra-Physics, Milpitas, CA, USA) while simultaneously being incubated at typical culture conditions of 37 °C with 5% CO_2_. Images (512 × 512 pixels; 1.05 × 1.05 μm/pixel X/Y resolution) were taken with a 25×, 1.1 numerical aperture (NA) objective (Olympus, Waltham, MA, USA) with a dwell time of 0.8 µs. Pixelwise optical redox ratios (ORR) were calculated by obtaining NADH (755 nm excitation/460 nm emission) and FAD (855 nm excitation/525 nm emission) two-photon excited fluorescence (TPEF) and then normalizing collected intensities to known concentrations of fluorescein [31]. Additionally, collagen SHG (855 nm excitation/427.5 nm emission) from the hydrogel was also collected to confirm thick collagen fibril formation in the hydrogels. Image analysis was performed using MATLAB version R2026b. ORR was calculated using Equation (1):

ORR = FAD/(FAD + NADH)(1)

### 2.8. Seahorse ATP Rate Assay

A Seahorse XF96 plate was used to culture cells for a total of 48 h. Cells (10,000 cells per well) were allowed to adhere to the surface of the plate for 24 h, followed by another 24 h in treated media. The cartridge was hydrated in XF Calibration solution the day before the experiment. Assay was run following the manufacturer’s protocol. Data were normalized to cell count using Hoechst stain.

### 2.9. Quantification of Extracellular Glutamine, Glutamate, and Lactate

Glutamine and glutamate were measured with Glutamine/Glutamate-Glo Assay (Promega, Madison, WI, USA, catalog #J8022) according to manufacturer’s instructions. Briefly, on day 6 of culture, media was removed and cells were washed with 1× PBS three times to remove residual media and drugs. Fresh media was added to the culture and to blank wells followed by a 24-h incubation period. Samples were collected and diluted 1:100 in 1× PBS to fall within the linear range of the assay. Two reactions were run simultaneously, with and without glutaminase, to determine total glutamine plus glutamate and glutamate alone. Samples were incubated for 60 min at room temperature and luminescence was measured. Glutamine was calculated by subtracting the glutamate only value from the total. L-lactate was measured with the L-lactate assay kit (Colorimetric) (Abcam, Waltham, MA, USA, catalog #ab65331) according to the manufacturer’s instructions. Samples were prepared similarly to glutamine measurements, following incubation of the assay, samples were measured for absorbance at 450 nm. For all samples, DNA was isolated and analyzed using Nanodrop for normalization.

### 2.10. Luminex Multiplex Assay

The day before harvesting, basal media were added to all hydrogels and incubated overnight. Conditioned media were subsequently collected and concentrated using Amicon Ultra 3 kDa molecular weight cutoff centrifugal filters. Luminex multiplex analysis (Bio-Techne, Minneapolis, MN, USA, catalog #LXSAHM) was performed to quantify BDNF, GDNF, NGF, and NT-3. DNA was isolated and analyzed using Nanodrop for normalization.

### 2.11. Dorsal Root Ganglia Culture

All procedures were approved by the University of Arkansas Institutional Animal Care and Use Committee (Animal Use Protocol #24018). The 3-week-old male and female Sprague Dawley rats (Envigo, Indianapolis, IN, USA) were used to isolate dorsal root ganglia (DRG) as previously established [28,32,33]. Rats were euthanized via carbon dioxide asphyxiation followed by decapitation. Afterwards, rats were shaved to expose the skin from the base of the tail to the top of the neck. Surgical scissors were used to cut the skin along the spine. Then, bone cutters were used to dislocate the cervical and lumbar regions of the spine. Once extracted, a medial dissection was performed, and spinal columns were placed in 1× PBS when not in use. The spinal column was exteriorly cleaned using surgical scissors to remove excess muscle. Using surgical scissors, the dorsal and anterior sides of the spinal column were cut medially to expose the spinal cord. Spinal cords were removed to reveal DRGs. Using forceps, DRGs were removed and cleaned using a scalpel. DRGs were stored in Hibernate-A media (Thermo Fisher, catalog #A1247501) until ready for dissociation. 

Isolation of sensory neurons was done following a previously established protocol [34]. Briefly, whole DRGs were subjected to media washes between 0.05% Trypsin (15 min at 37 °C) and 0.1% collagenase 1-AS (90 min at 37 °C). Following 1 h suspension in tissue culture flask, supernatant was collected and neurons were seeded at 20,000 cells per gel. Neurobasal media (Thermo Fisher, catalog #10888022) containing 10% FBS, 1% P/S and 2% B27 supplementation was used for neuron cultures. No β-NGF was supplemented in any cultures. Equal volumes of media were added in multi-cell cultures. For conditioned media experiments, neurons were seeded on top of blank collagen gels for 24 h. Following, addition of antibody solutions: rat anti-GFR alpha-1 (4 µg/mL; Bio-Techne, catalog #AF560) and goat anti-TrkB (1 µg/mL; Bio-Techne, catalog #AF1494), or goat IgG isotype control (4 µg/mL; Bio-Techne, catalog #AB-108-C), with 20× concentrated conditioned media from a single MCF cell culture was added to neuron media for 48 h.

### 2.12. Immunofluorescence Staining

Hydrogel cultures were fixed in 3.7% formaldehyde (Sigma Aldrich, catalog #252549) for 1 h at 4 °C and subsequently rinsed 3 times with PBS at room temperature (RT). Cultures were permeabilized with 0.05% Triton X-100 (Sigma Aldrich, catalog #X100) in PBS for 7 min and then blocked with 1% bovine serum albumin (BSA; Sigma Aldrich, catalog #A7906) in PBS for 1 h at RT. Primary mouse anti-neurofilament (1:400; Developmental Studies Hybridoma Bank (DSHB), Iowa City, IA, USA, catalog #RT97) antibodies were added to fresh 1% BSA and incubated with samples overnight at 4 °C. Cultures were washed with 0.05% Tween-20 (Sigma Aldrich, catalog #P9416) in PBS 3 times at RT. Next, goat anti-mouse AlexaFluor 488 (1:500; Fisher Scientific, catalog #2610355) secondary antibody, rabbit anti-phalloidin (1:400; Fisher Scientific, catalog #A22283) and DAPI (1:2500; Thermo Fisher Scientific, catalog #D1306) were added to 1% BSA and incubated with samples for 1 h at RT. Finally, samples were washed twice with 0.05% Tween-20 and once with PBS before storage in fresh PBS at 4 °C protected from light until ready for imaging. Microscopy was conducted using the Olympus IX-83 Automatic Inverted Confocal Microscope using 4× magnification and 1× digital zoom.

### 2.13. Outgrowth Analysis

Volumetric images of neuron specific marker, neurofilament, were compressed into a single slice using FIJI (ImageJ) version 1.54p z-project function, at max intensity, across all slices. A custom MATLAB code was written to automatically identify neuron cell body (soma) and neurites. Soma identification was performed via morphological characterization first by applying a Gaussian filter and thresholding neuron regions. Candidate soma was confirmed if diameter was between 10 and 30 μm and with circularity of at least 0.5. Neurite segments were identified following thresholding of neurofilament channel to remove unspecific noise. Following thresholding, the mask was skeletonized and measured for total length. Soma regions were excluded from the neurite skeletonized length measurements. Mean outgrowth was calculated by quantifying total neurite length per field of view and averaged based on soma count. MATLAB code was generated in part with assistance from Claude. All code was reviewed and tested by the authors.

### 2.14. Statistical Analysis

Groups were compared using tests for significance as indicated in the figure legends. All data are presented as mean ± standard deviation where *p* < 0.05 was considered statistically significant. GraphPad Prism version 10.6.1 was used to present data as mean ± standard deviation.

## 3. Results

### 3.1. Transcriptomic Profiling Highlights Early-Stage Specific Metabolic Regulation

**Figure 1:**
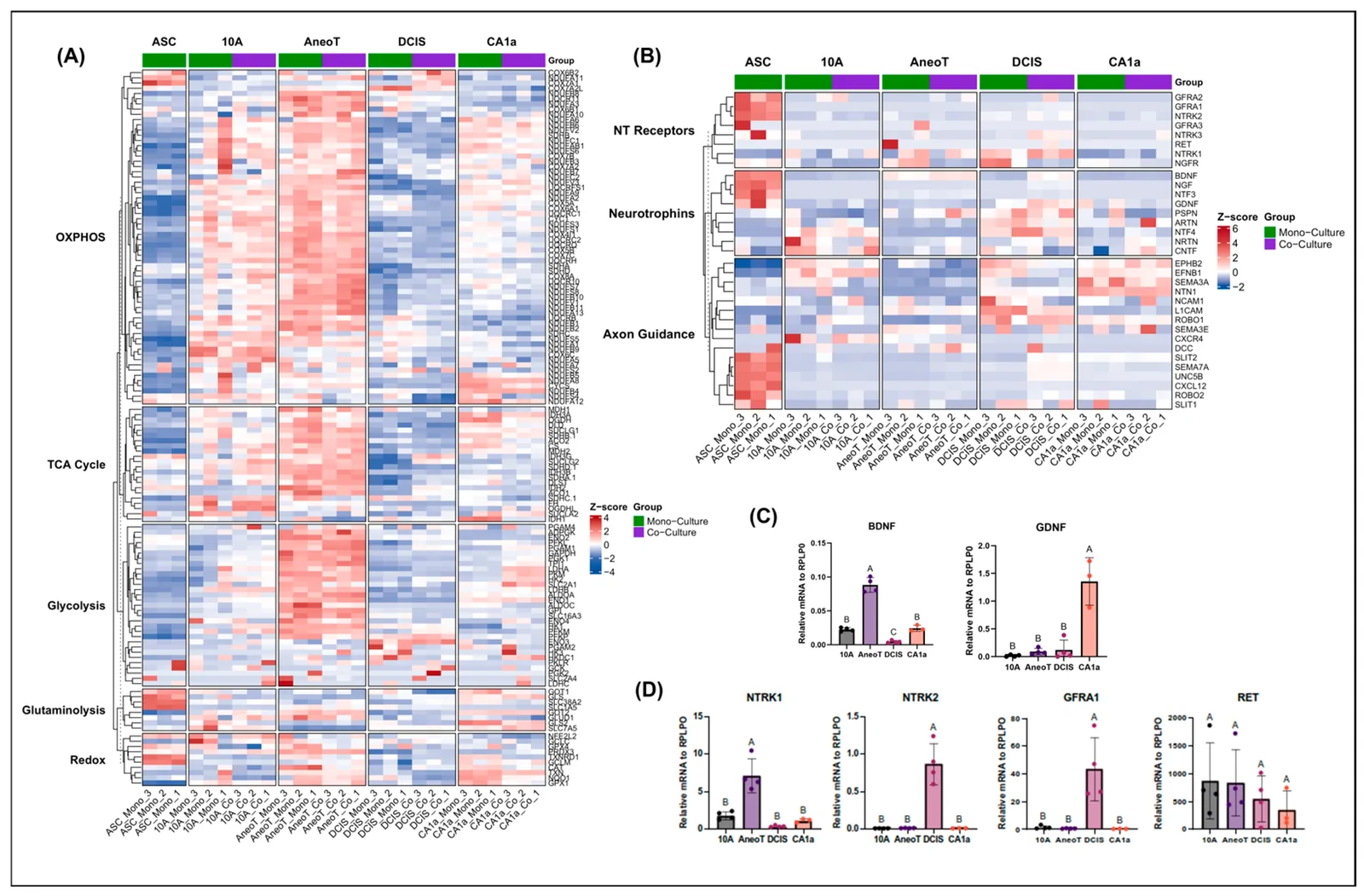
Bulk RNA-sequencing. (**A**) Hallmark metabolism associated genes for OXPHOS, TCA cycle, glycolysis, glutaminolysis, and redox. (**B**) Heatmap of neurotrophins, neurotrophin receptors, and hallmark axon guidance. N = 3. Validation of bulk RNA-seq results via qPCR on (**C**,**D**) BDNF, GDNF, NTRK1, NTRK2, GFRA1, and RET. One-way ANOVA with Tukey’s post hoc comparison. Different lettering indicates significant differences (*p* < 0.05). N = 3–4.

To obtain a comprehensive understanding of our MCF10 panels with or without ASC cocultures in a tumor-mimetic environment, 3D monocultures of MCF10 series or coculture with ASCs were established using cold-cast collagen hydrogels [28,29]. After 5 days, these cultures were subjected to bulk RNAseq. Compared with normal 10A cultures, AneoT cells had overall elevated metabolic regulation including glycolysis, OXPHOS, and TCA cycle (Figure 1A). Interestingly, these sharp increases were somewhat attenuated in DCIS.com and CA1a cultures, although cocultures with ASCs exhibited elevated metabolic gene signatures. ASC monocultures exhibited elevated expressions of neurotrophins including BDNF, NGF, NTF3, and GDNF as well as their receptors NTRK2, GFRA1, GFRA2, and GFRA3. ASCs also expressed the hallmark axon guidance genes: SLIT1, SLIT2, SEMA7A, UNC5B, ROBO2, and CXCL12 (Figure 1B). Moreover, our qPCR results also showed increases in BDNF and NTRK1 expression in AneoTs, NTRK2 and GFRA1 in DCIS, and GDNF in CA1a (Figure 1C,D).

### 3.2. Optical Redox Imaging Reveals Cell Stage-Specific Responses to Metabolic Inhibitors

**Figure 2:**
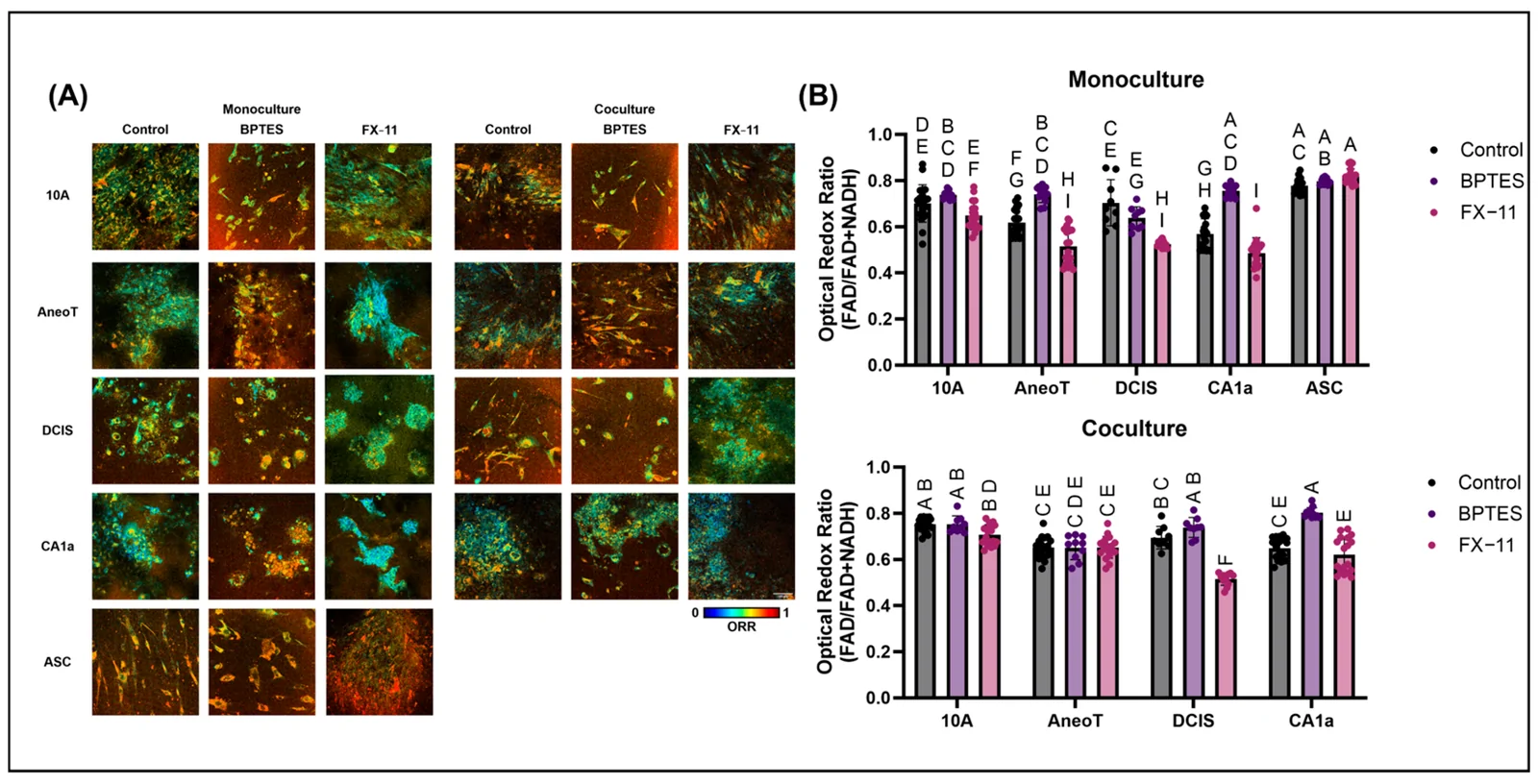
MCF10 cell metabolic characterization via MPM optical redox imaging. (**A**) Representation of MPM optical redox images of MCF10 cell series in mono- and ASC cocultures. Scale Bar: 100 µm. Quantified optical redox ratio in (**B**) monoculture and coculture platforms with BPTES and FX-11 treatments. Each dot represents mean cell optical redox ratio (ORR) in a field of view. Two-way ANOVA with Tukey’s post hoc comparison. N = 3–6. Different lettering indicates statistical significance (*p* < 0.05).

To characterize metabolic behavior across the MCF10 isogenic progression series, we performed two-photon excited fluorescence optical redox imaging using multiphoton microscopy based on endogenous autofluorescence of NADH and FAD and calculated ORR. As the cell line malignancy increases, we observed a decrease in ORR (Figure 2A,B), consistent with a more reduced environment where NADH accumulation occurs relative to FAD as cells advance towards malignancy [35]. To test whether glutaminolysis was driving this reduced state, we inhibited GLS1 with a pharmacological allosteric inhibitor BPTES. Treatment resulted in a significantly increased ORR in AneoT and CA1a cultures (Figure 2B), contributing to overall oxidation of the intracellular environment. Simultaneously, 10A, DCIS, and ASCs were unaffected by treatment (Figure 2A,B).

To complement glutaminolysis blockade, we targeted glycolytic restoration of NAD^+^ by inhibiting LDHA with a pharmacological inhibitor FX-11. In contrast to BPTES, FX-11 treatment significantly decreased ORR across the MCF10 progression series (Figure 2A,B), which is consistent with the accumulation of NADH in the cytosolic region following a decrease in pyruvate to lactate conversion and NAD^+^ regeneration. In monocultures, FX-11 significantly decreased the ORR of AneoT, DCIS, and CA1a. FX-11 had no effect on ORR results in 10A or ASC cultures.

### 3.3. Seahorse ATP Rate Assay Shows Cell Type- and Inhibitor Response-Specific Preferences to ATP Production Using Glycolysis Versus Oxidative Phosphorylation

**Figure 3:**
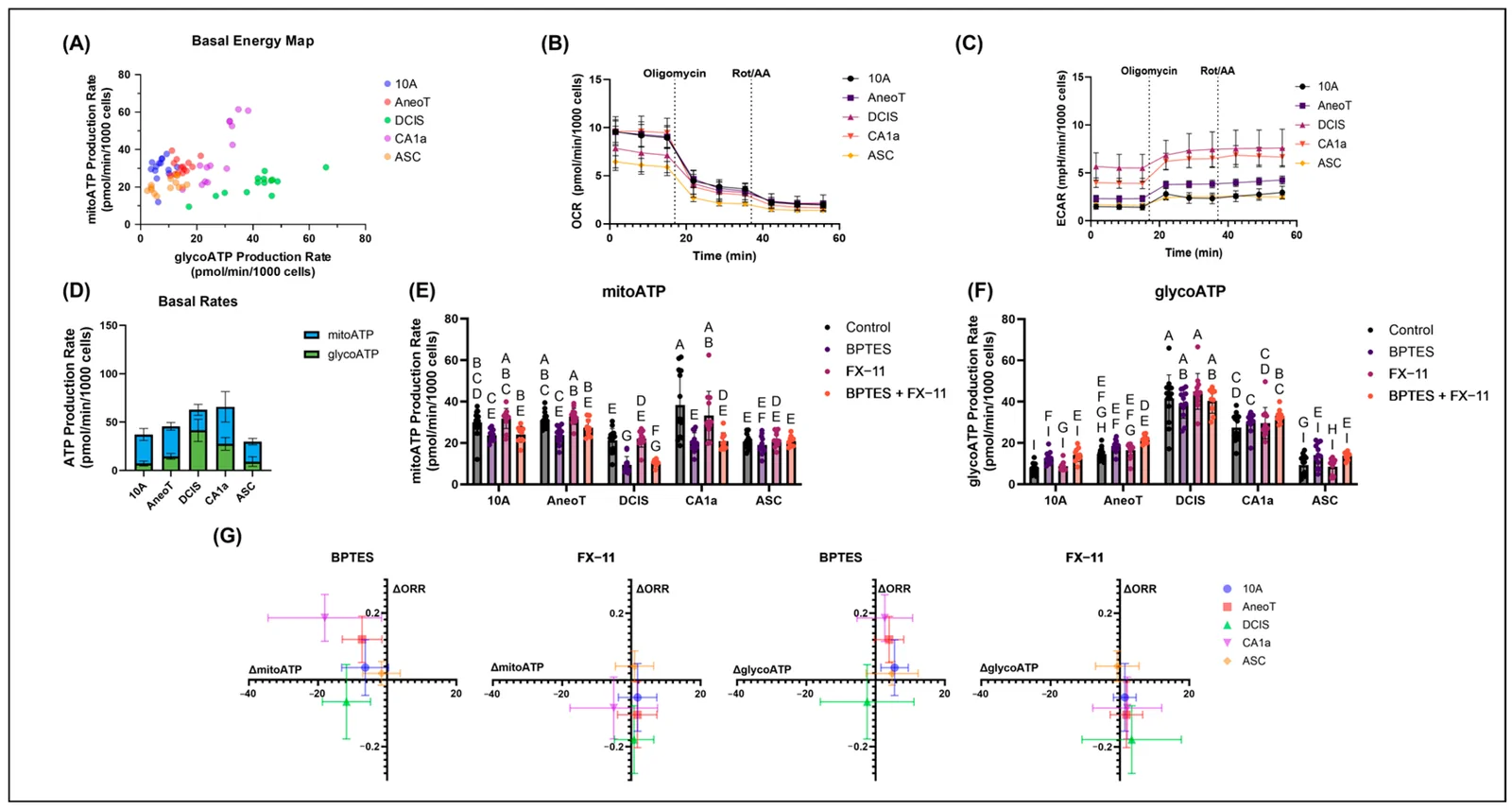
Seahorse ATP rate analysis suggests metabolic inhibition with BPTES but not FX-11. (**A**) Basal energy scatter map measured by Seahorse ATP rate assay on MCF cells. (**B**) Oxygen consumption rate (OCR) and (**C**) extracellular acidification rate (ECAR) representations containing oligomycin and rotenone Antimycin A (Rot/AA) injection. (**D**) Basal energy rate of glyco- and mitoATP stacked bar graph. (**E**) Mito- and (**F**) glycoATP bar graph representing all MCF10 cells and treatments across experiments. Two-way ANOVA with Tukey’s post hoc test. Different lettering indicates statistical significance (*p* < 0.05). N = 3. (**G**) Scatter plot representation of independent MPM and Seahorse experiments.

Seahorse ATP Rate assay was performed to directly measure ATP production preferences using glycolysis versus OXPHOS. Analysis of total ATP revealed increasing basal energetics across cell lines, where DCIS and CA1a exhibited the overall highest ATP production rates (Figure 3A–D). DCIS cells had the highest contributing ATP generation via glycolysis, followed by CA1a retention of elevated glycolytic rates compared to 10A and AneoT. Yet, mitoATP production of CA1a was significantly higher than DCIS (Figure 3E,F). Consistent with ORR data, Seahorse ATP rate assay revealed that BPTES treatment suppressed mitochondrial ATP synthesis in DCIS and CA1a. On the other hand, FX-11 had minimal effects on all cultures (Figure 3C,D). This suggests that while FX-11 was successful in the blockade of LDHA for redox balancing, it garnered no significant influence on either glycolysis or mitochondrial metabolisms.

### 3.4. Extracellular Metabolites Reveal Stage-Specific Inhibition Capacities of BPTES and FX-11

**Figure 4:**
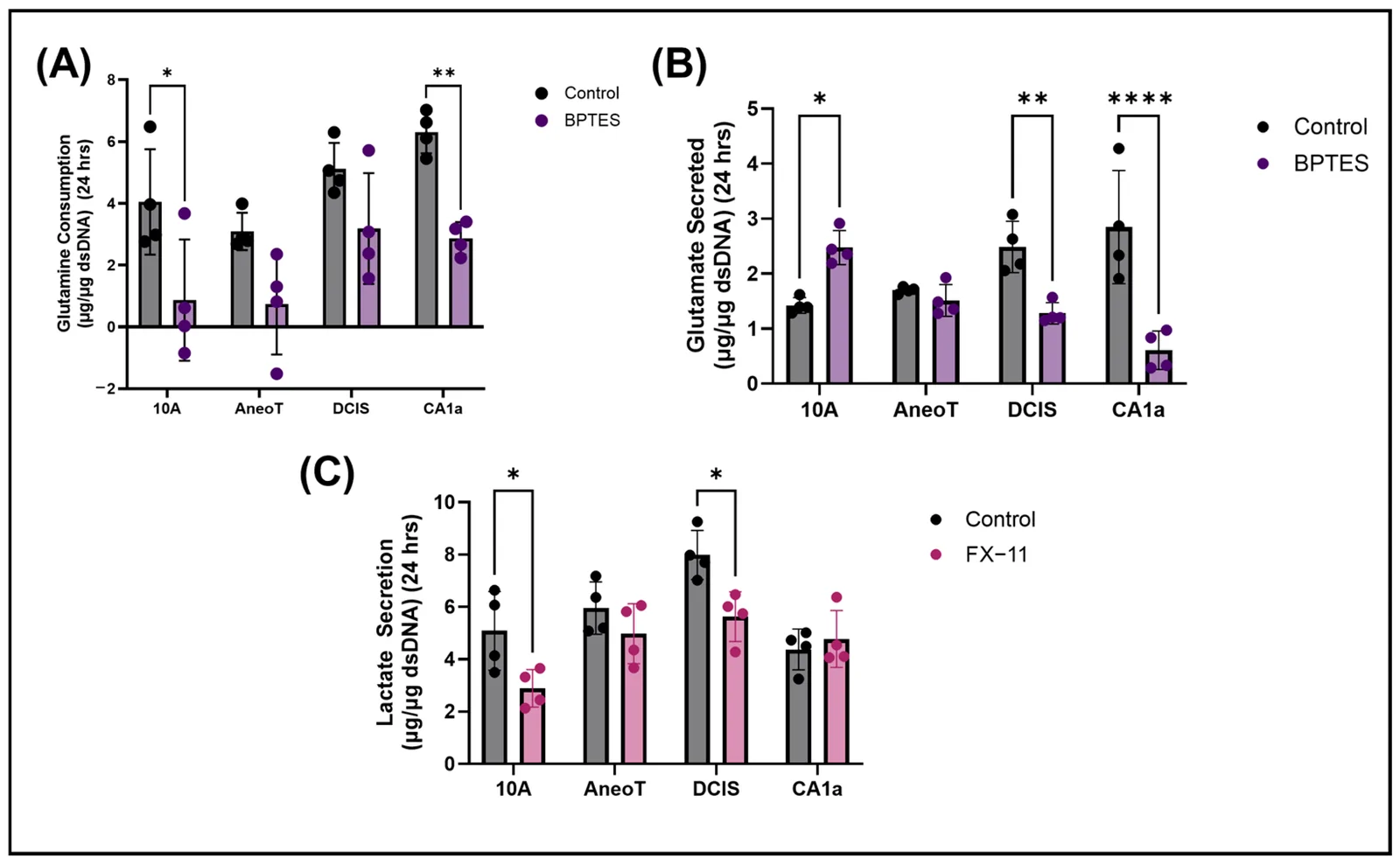
Extracellular metabolite measurements of media following 24-h incubation. (**A**) Glutamine consumption with BPTES inhibition, (**B**) glutamate secretion with BPTES inhibition and (**C**) lactate secretion with FX-11 inhibition. Two-way ANOVA with Tukey’s post hoc test between treatment only: * *p* < 0.05, ** *p* < 0.01, **** *p* < 0.0001. N = 4.

To evaluate the effect of glutaminolytic and glycolytic flux and the efficacy of BPTES and FX-11 on targeting glutaminolysis and lactate secretion, respectively, direct metabolite measurements of culture media were performed. As MCF10 cells progress, they increasingly consume glutamine and secrete glutamate. By blocking GLS1 with BPTES, there is a significant reduction in consumption of glutamine and secretion of glutamate from CA1a (Figure 4A,B). In contrast, DCIS cells generated the bulk of the ATP via glycolysis and had the highest overall secretion of lactate; it was observed that FX-11 significantly reduced the secretion of lactate (Figure 4C).

### 3.5. Neurotrophin Switch Aligns with Progression and Metabolic State

**Figure 5:**
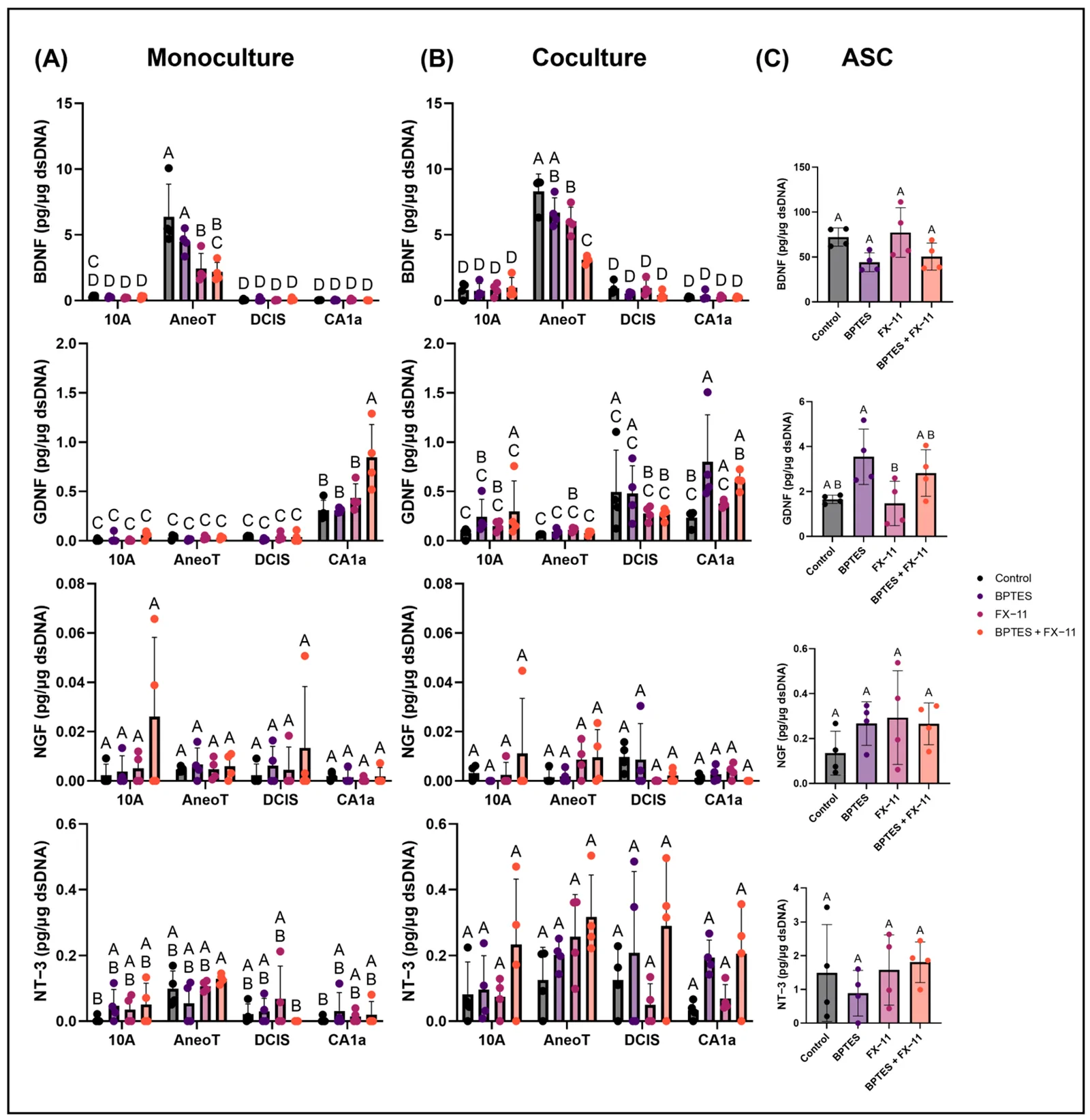
MCF10 cell series expresses distinct neurotrophin secretion profile of BDNF, GDNF, NT-3, and NGF in (**A**) monocultures (**B**) cocultures and (**C**) ASC-only cultures. Two- and one-way ANOVA with Tukey’s post hoc test. Different lettering indicates statistical significance (*p* < 0.05). N = 4.

Neurotrophins were measured to evaluate how specific MCF10 cell lines with or without ASC cocultures release neurotrophic factors BDNF, NGF, GDNF, and NT-3 in a tumor-mimetic environment. Healthy, parental 10A cells showed minimal detectable neurotrophin secretion. In contrast, AneoT cells showed a strong release of BDNF compared to all other neurotrophins measured. Similar to 10A, DCIS cells produced minimal detectable neurotrophin secretion. In contrast, CA1a cells displayed a switch, favoring GDNF expression over any preference towards BDNF, NGF, and NT-3. Minimal expressions of both NT-3 and NGF were detected across all MCF10 series (Figure 5A). Of all five cell types tested, ASCs had minimal additive effect when in direct coculture with MCF10 cells (Figure 5B), yet secreted the highest overall amount of neurotrophins when cultured alone (Figure 5C). To probe mechanistic behavior behind the preferential neurotrophin release in AneoT and CA1a cells, these cultures were treated with BPTES, FX-11, as well as a combination of the two drugs. AneoT cultures showed a significant drop in BDNF when treated with FX-11. Interestingly, while BPTES and FX-11 independent treatments had negligible effects on GDNF secretion rates in CA1a cultures, combination therapy significantly increased GDNF secretion in monoculture platforms (Figure 5A). In coculture platforms, AneoTs exhibited similar behavior to monocultures, whereas CA1as increased GDNF release, suggesting ASC additive effects on GDNF secretion. Notably, these inhibitors had minimal effects on ASC secretion of all neurotrophic factors tested (Figure 5C).

### 3.6. Early-Stage AneoT Display Superior Neurite Outgrowth Capacity In Vitro

**Figure 6:**
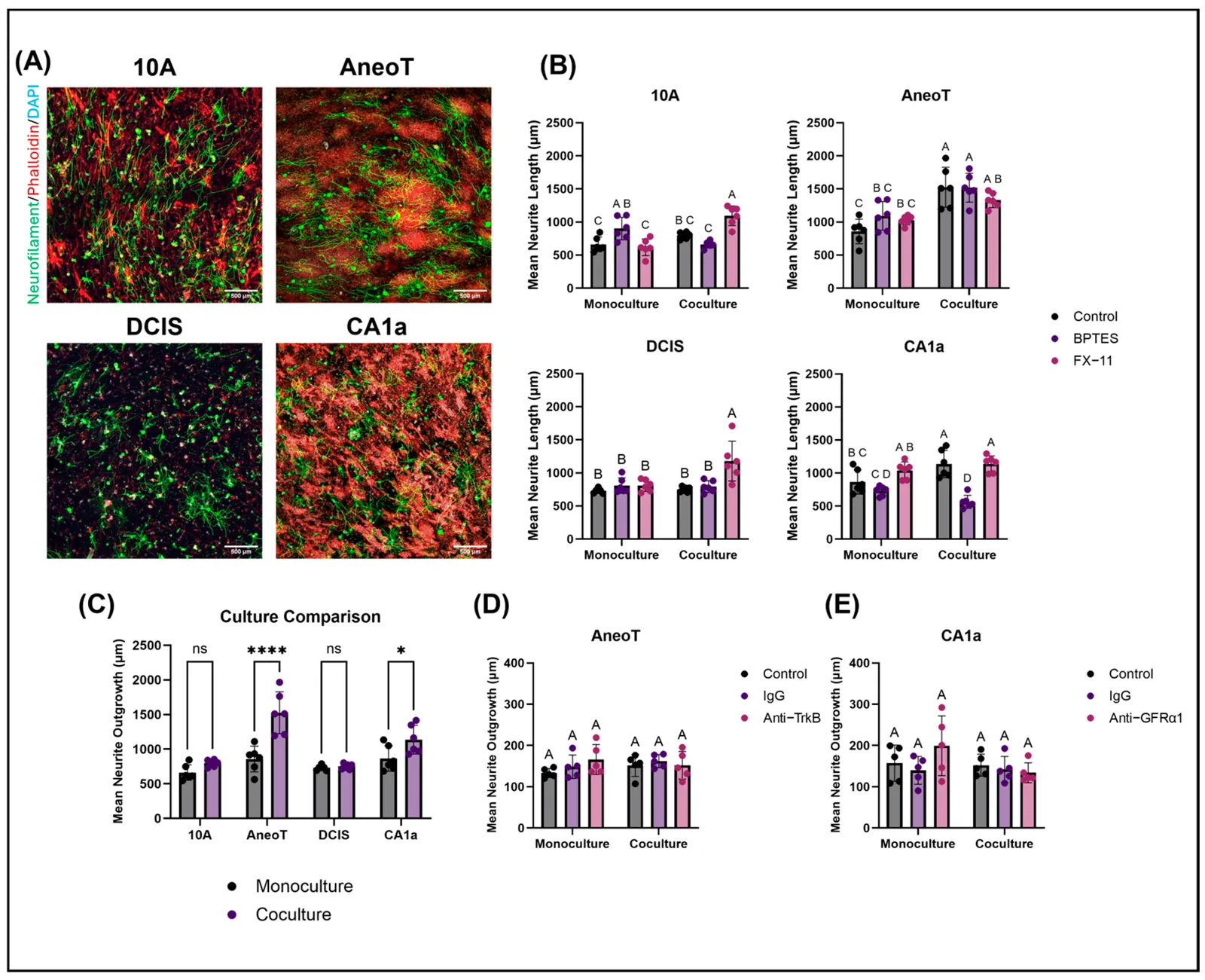
DRG neurite outgrowth in vitro. (**A**) Composite immunofluorescence images of coculture platform with isolated neurons, F-actin (red), neurofilament (green) and DAPI (cyan). Scale Bar = 500 µm. (**B**) Mean DRG neurite outgrowth from MCF10 series mono- and ASC cocultures with or without BPTES or FX-11 treatment. (**C**) Mean DRG neurite outgrowth from MCF10 series mono- and ASC cocultures. Two-way ANOVA with Tukey’s post hoc test between mono and coculture only: ns: not significant; * *p* < 0.05, **** *p* < 0.0001. N = 6. (**D**) Mean neurite outgrowth from AneoT and (**E**) CA1a conditioned media with IgG control and function blocking BDNF and GDNF, respectively. Two-way ANOVA with Tukey’s post hoc test: Different lettering indicates significant differences (*p* < 0.05). N = 5–6.

**Figure A1:**
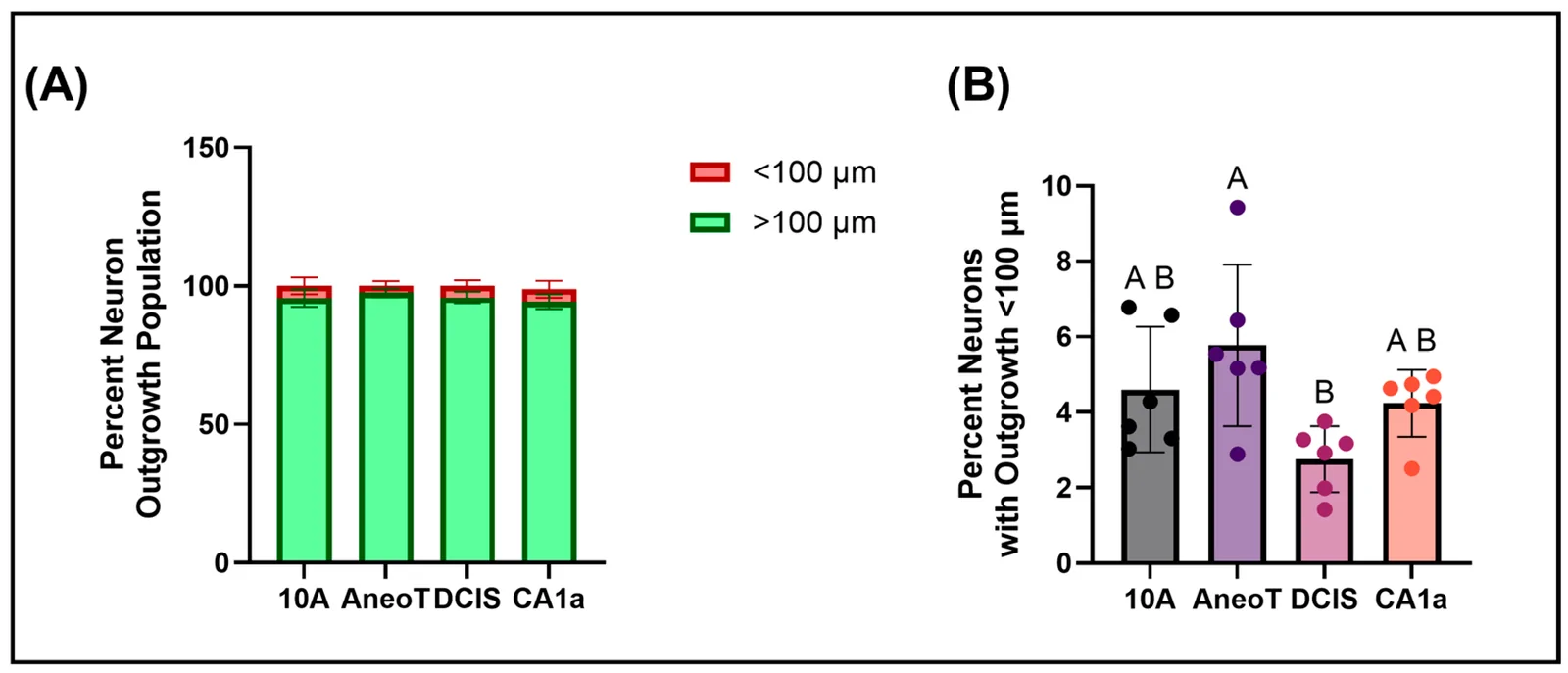
Direct culture sensory neuron outgrowth distance population. (**A**) Stacked percentage plot and (**B**) bar graph representation of percent neurons with outgrowth < 100 µm. One-way ANOVA with Tukey’s post hoc comparison. Different lettering signifies significant differences (*p* < 0.05). N = 6.

To assess tumor innervation propensity of cancer cells, sensory neurons were isolated from 3-week-old Sprague Dawley rat DRGs and were seeded atop MCF10 mono- or ASC cocultures (Figure 6A). Neurite outgrowth was measured by the total measurable neurites and normalized to soma count. All cultures induced DRG neurite outgrowth in vitro. Minimal impact was evident following treatment with BPTES and FX-11 (Figure 6B), except in CA1a + ASC cocultures where BPTES had significantly reduced neurite outgrowth. In addition, DCIS + ASC cocultures treated with FX-11 presented with greater neurite outgrowth. The overall magnitude of neurite outgrowth in monocultures had no significant difference; however, in ASC cocultures, AneoT and CA1a had significantly higher DRG neurite outgrowth (Figure 6C). Similarly, direct culture contained over 90% of sensory neurons with neurites of more than 100 µm (Figure A1). To investigate the role these neurotrophic profiles have on neurite outgrowth independently, we tested conditioned media alongside function-blocking antibodies. Here, we see an overall reduction in outgrowth capacities when comparing direct culture to indirect cultures as well as no differences in outgrowth when blocking receptors for both BDNF (TrkB) and GDNF (GFRα1) interactions with neurons (Figure 6D,E).

## 4. Discussion

In this study, we sought to investigate how metabolic rewiring across different stages of breast cancer cells directly contributes to breast tumor innervation. First, we characterized distinct metabolic trajectories across isogenic cell lines via RNA sequencing, optical redox imaging, and functional bioenergetic profiling through ATP rate measurements. Our findings identified the glutamine-oriented metabolism by late-stage breast cancer cells contributing to redox homeostasis, TCA anaplerosis, and sustained oxidative metabolic capacity. AneoT cells, characterized with early onset of H-ras mutation, exhibited early transcriptional upregulation of glycolytic, TCA, and OXPHOS genes, suggesting a shift towards more energetic phenotypes. Interestingly, translational effects in DCIS cells indicate elevated glycolytic function while CA1a show elevated glycolysis but acquire glutaminolysis flexibility. Our results corroborate prior literature with elevated glycolysis in transition to DCIS [36,37].

**Figure A2:**
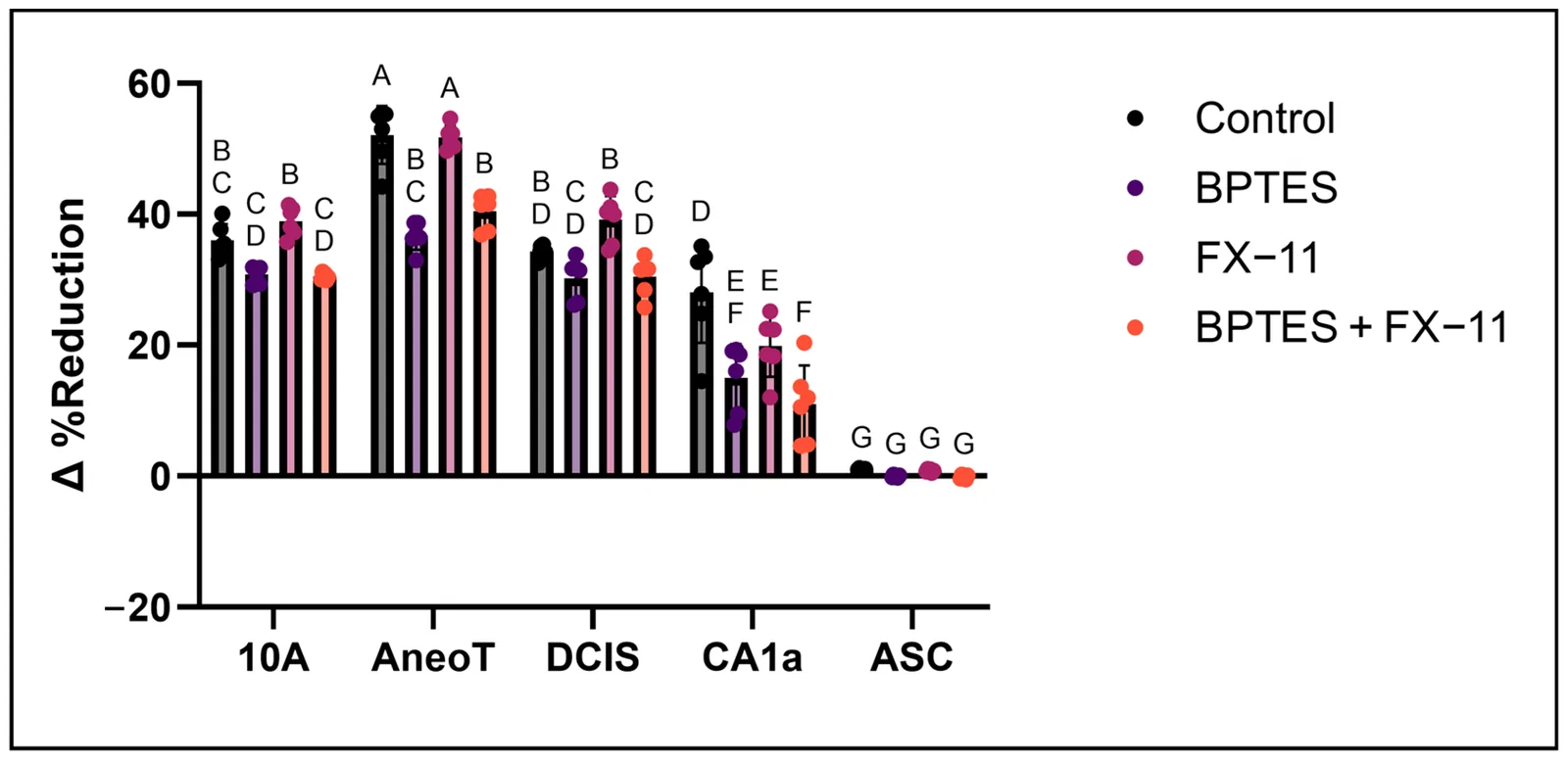
AlamarBlue results across MCF10A cell series and ASC monoculture. Two-way ANOVA with Tukey’s post hoc comparison. Different lettering indicates significant differences (*p* < 0.05). N = 6.

**Figure A3:**
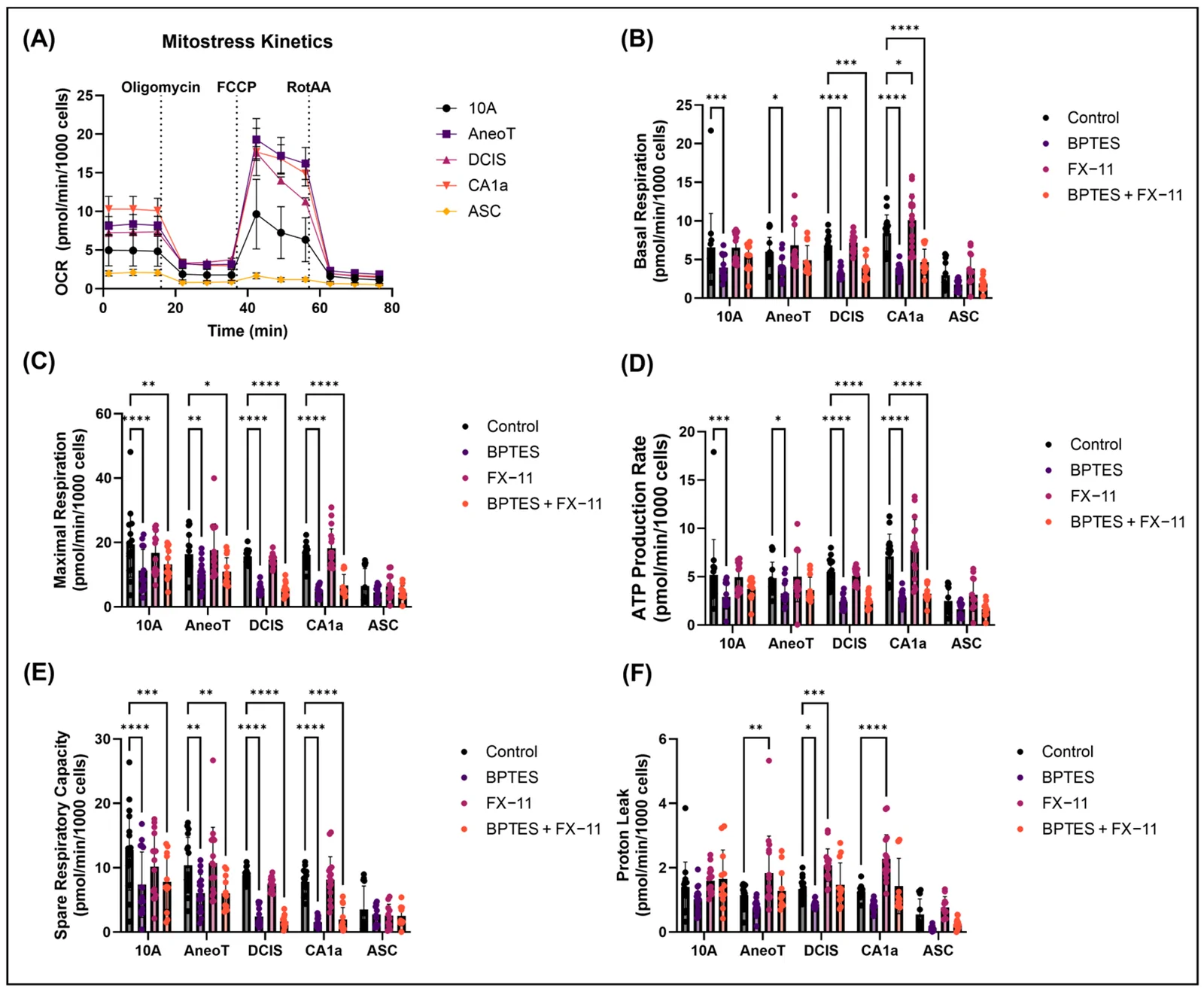
Seahorse Mitostress results. (**A**) Basal OCR Kinetics, (**B**) basal respiration, (**C**) maximum respiration, (**D**) ATP production, (**E**) spare respiratory capacity, and (**F**) proton leak. Two-way ANOVA with Tukey’s post hoc test between treatments: ns: not significant; * *p* < 0.05, ** *p* < 0.01, *** *p* < 0.001, and **** *p* < 0.0001. N = 3–4.

Within the last decade, researchers have implemented multiphoton microscopy to measure tumor autofluorescence of metabolic co-factors NADH and FAD in vitro, in vivo, and in clinical applications of cancer research [38,39]. ORR declines as cellular redox becomes more reduced, a state typically associated with elevated glycolysis, e.g., NAD^+^ consumption at glyceraldehyde-3-phosphate (G3P) and regenerating NADH through LDH [40]. Subsequent reduction from the electron transport chain and generation of ATP via OXPHOS is also a major contributor, particularly by supplementing the TCA cycle with glutamine with cancer progression and metastasis [41,42]. By blocking GLS1 with BPTES, we demonstrated that AneoT and CA1a relied on glutamine as a source of NADH and a supplemental metabolite to fuel mitochondrial metabolisms. Similarly, in AneoT and CA1a, alamarBlue assay revealed a significant reduction in metabolic function when treated with BPTES (Figure A2). Further evidence suggestive of CA1a reliance on glutamine was evaluated via Seahorse Mitostress test, which indicated that CA1a had the lowest level, and most reduced, spare capacity in the progression series (Figure A3). This, in conjunction with CA1a consuming significantly more glutamine with the largest reduction with BPTES, describes GLS1-mediated glutaminolysis for TCA cycle and OXPHOS. In contrast, FX-11-mediated inhibition of LDHA resulted in the opposite, suggesting a blockade of NAD^+^ regeneration and NADH pooling in the more glycolytically active cells compared to both normal mammary epithelial and stromal cells.

To further corroborate metabolic distinctions among progressed cells, ATP rate assay was used to measure preferential generation of ATP between glycolysis and mitochondrial metabolisms. DCIS cultures generated most of their ATP through glycolysis, even to a greater extent than CA1a cells. Indeed, formation of traditional Warburg-like cells has been previously attributed to DCIS where a transition to metastatic phenotypes relies on glutamine consumption [36,37]. Despite an elevated mitochondrial oxygen consumption rate in AneoT, CA1a utilized glutaminolysis for metabolic regulation, aligned with previous literature [43]. Beyond bioenergetics, glutamine supports the replenishment of the TCA cycle, redox homeostasis via glutathione, and has implications in therapeutic resistance and metastatic progression [44,45,46]. Conversely, we observed no significant differences in ATP production upon FX-11 treatment; this indicates that LDHA-dependent reduced states generated through the conversion of pyruvate to lactate did not greatly alter mitochondrial metabolism or function.

Notably, the lack of response to inhibitor treatment by MCF10A and ASC cultures signaled metabolic independency from glutamine in maintaining stable NADH production and LDHA in recycling NAD^+^. This agrees with current literature on metabolic adaptability of mesenchymal stem/stromal cells (MSCs) including ASCs [47]. Interestingly, recent studies have shown that LDHA inhibition can attenuate immunoregulatory properties of MSCs [48]. This poses an interesting yet complex question into the validity of LDHA inhibition in cancer therapies, as tumor-secreted lactate is known to facilitate an immunosuppressive environment [49]. Current investigations into the capacities of FX-11 on cancer metabolisms differ; despite the understanding that FX-11 targets cytosolic NAD^+^ generation, its mechanism is dissimilar to other forms of LDHA inhibitors [50,51]. Similar methodological approaches have suggested that FX-11 may act as a mitochondrial uncoupler in melanoma cells [52], while in other cancers the oxidative stress generated results in mitochondrial ATP loss [53]. In investigating the modality FX-11 has on MCF10 cells, mitostress evaluation shows features suggesting FX-11 may behave as a mitochondrial uncoupler as we observe elevated proton leak (Figure A3). We, however, observe the capacity of FX-11 to reduce ORR and target lactate secretion of glycolytic DCIS although having no effect on overall glycoATP production on any cells included in our study. By reducing overall ORR, FX-11 results indicate a shift in available cytosolic pool of NAD^+^, reflected by NADH accumulation via G3P. Rather than targeting ATP supply directly, NAD^+^ redox-mediated shift could represent the principal consequence of LDHA inhibition in MCF10 series, while not impacting glycolysis. The intricate balance of LDHA inhibition effects on cancer cells versus stromal cells along with subsequent effects on tumor innervation is yet to be clearly elucidated. Notably, FX-11 treatment produced a modest, but significant, increase in neurite outgrowth from 10A and DCIS, indicating that LDHA inhibition may inadvertently create a more axonal-friendly environment across different stages of the disease. This effect was confined to lines with lower baseline outgrowth and neurotrophic factors but where significant impact was observed on lactate secretion, suggesting it is distinct from neurotrophic factor-driven observations, warranting further investigation.

Neurotrophins are growth factors that regulate the development, maintenance, and function of the central and peripheral nervous systems. GDNF is known to promote synaptic terminal formation, myelination, and neural stem and progenitor cell migration and proliferation, while NGF fosters glial survival and neurite outgrowth [54,55,56]. NGF is known to be over-expressed in breast tumors and notoriously benefits tumor progression by recruiting neurites to the TME [57]. Finally, NT-3 signaling regulates peripheral nerve outgrowth following injury and, in breast cancers, it is elevated compared to healthy individuals and contributes to brain metastasis [58,59]. In general, BDNF facilitates axonogenesis, glial survival, and ASC migration and proliferation, and in breast cancer BDNF levels correlate with breast tumor progression [60,61]. Moreover, BDNF supplementation promoted lactate production and glucose uptake in neurons and regulated via HIF1α [62,63]. Similarly, in cancer-associated stromal cells, lactate is taken up by MCT4, inducing stress response NF-κB-mediated upregulation of BDNF [64]. Here, however, FX-11 is not reducing BDNF in tandem with reduced lactate production in AneoT. A possible alternative mechanism where redox homeostasis, specifically NAD^+^ depletion, may be contributing to BDNF expression, similar to that seen in neuron-derived BDNF secretion [65]. In the present study, we found distinct neurotrophin profiles expressed across MCF10 cell series, where AneoT cells had elevated BDNF secretion while CA1a cells showed a marked increase in GDNF release. Interestingly, FX-11 treatment reduced BDNF from AneoT while a combination approach, BPTES and FX-11, targeting both metabolite resources and redox homeostasis, resulted in elevated GDNF in CA1a cultures. AneoT cells were overall more sensitive to both inhibitors, as demonstrated by a significant drop in BDNF release. In contrast, CA1a was overall less responsive, and even elevated GDNF release when either BPTES or FX-11 were added. Conditioned media alone, however, did not significantly contribute to axonogenesis when cancer–neuron interactions were removed, suggesting neurotrophin production plays a more supporting role like survival or maintenance. In addition, outgrowth may depend on direct cell–cell interactions, for example, through NCAM, L1CAM, and N-cadherin (CDH2) (Figure 1B). Alternatively, the 3D collagen platform used in the present study may sequester secreted factors in higher concentrations within the bulk of the gel, where conditioned media evaluation represents a smaller fraction and less localized neurotrophin population. This may contribute to the weaker responses in the conditioned media counterpart to direct culture. Further studies focusing on cell–cell and cell–ECM, ECM remodeling, and how GDNF and BDNF support sensory neurons will be needed to define how tumor cells establish and maintain a pro-axonogenic niche.

While our RNAseq data showed most elevated metabolic signatures from AneoT cultures, measured flux revealed DCIS as the most glycolytic while AneoT were also most sensitive to metabolic inhibitors in terms of BDNF release but not towards FX-11 prevention of lactate secretion. This apparent discrepancy likely reflects transcriptional capacity and metabolic flux, where in the context of metabolic markers, transcriptional signatures are better as a metabolic capacity rather than as a metabolic flux capability [66]. We also observed no difference in NGF or NT-3 secretion from all mono- and cocultures. Furthermore, while DCIS cultures showed very minimal neurotrophin release, even with ASC cocultures, these cells exhibited significantly higher transcription of BDNF and GDNF receptors. This suggests that DCIS may have a preference towards receptor-priming over neurotrophin release. Overall, this demonstrates an interesting biphasic behavior in neurotrophin and receptor expression across breast cancer stages, with early pre-malignant and aggressive cultures elevating neurotrophin release while DCIS cells primarily express neurotrophin receptors. The precise mechanisms behind cell type-specific neurotrophin release profiles in our study, as well as how the metabolic inhibitors altered transcription and translation of these growth factors, needs further elucidation.

When MCF10 mono- or ASC cocultures were overlaid with DRG sensory neurons, neurite outgrowth was observed in all cultures. Interestingly, AneoT and CA1a cultures showed elevated average DRG neurite outgrowths in their respective ASC cocultures. This behavior coincides with elevated BDNF secretion from AneoT and GDNF from CA1a cultures, implying that these two neurotrophic factors may contribute to extending neurite outgrowth. In addition, given the elevated neurite outgrowth observed in ASC cocultures, the specific effects of stromal cells in driving tumor innervation will need to be further elucidated. Retrospective and case studies have found early DCIS tumors to be significantly more innervated than healthy tissues, and innervation heightens with the progression of DCIS to invasive breast cancer [17,67]. Interestingly, mitochondrial transfer from neurites to cancer cells was recently shown to bolster mitochondrial oxygen consumption by cancer cells [68]. Future studies on how these neurites interact with cancer cells and how metabolic inhibition impacts these cell–cell communications will help unravel metabolic–axonogenic synergies driving tumor innervation and subsequent metastases.

## 5. Conclusions

Breast tumor innervation is a rapidly emerging contributor to disease aggravation, yet how the tumor microenvironment facilitates this process is largely elusive. In this study, we showed that MCF10 isogenic series of human mammary epithelial cells exhibit differential metabolic and axonogenic behaviors, which can be further potentiated by the presence of stromal cells. Stage-specific preferences in neurotrophin release were observed, which coincided with longer DRG neurite outgrowth. Taken together, these results offer a foundation for mechanistic investigation and suggest several feasible directions for future work: the specific effects of stromal cells on facilitating tumor innervation, how different neurotrophin landscapes drive neurite infiltration, and the combined role of metabolic and neurotrophic perturbations of breast tumor innervation. Further, probing ribosomal activity could help elucidate differences between the transcriptional and protein-level data observed.

## Figures and Tables

**Table 1:** Gene primer sequences containing forwards and reverse.

Gene	Forward	Reverse
RPLP0	ACACCTTCCCACTTGCTGAA	CTCCTCCGACTCTTCCTTGG
BDNF	TTTGGTTGCATGAAGGCTGC	GCCGAACTTTCTGGTCCTCA
GDNF	TCTTGCCCCTGACCTGTTG	ATCTTAAAGTCCCGTCCGGC
NTRK1	CACTAACAGCACATCTGGAGACC	TGAGCACAAGGAGCAGCGTAG
NTRK2	TCGTGGCATTTCCGAGATTGG	TCGTCAGTTTGTTTCGGGTAAA
GFRA1	CGGGCAATACACACCTCTGT	CTTGGAGGAGCAATTGA
RET	GAAAAGTGGTCAAGGCAACG	AAATCTTCATCTTCCGCCCC

## Data Availability

The original contributions presented in this study are included in the article. Further inquiries can be directed to the corresponding author.

## Abbreviations

The following abbreviations are used in this manuscript:

ASC	Adipose stromal/stem cell
BDNF	Brain-derived neurotrophic factor
DRG	Dorsal root ganglia
FAD	Flavin adenine dinucleotide
FBS	Fetal bovine serum
GDNF	Glial cell-line derived neurotrophic factor
GLS1	Glutaminase
LDHA	Lactate dehydrogenase
MSC	Mesenchymal stem/stromal cell
NA	Numerical aperture
NAD	Nicotinamide adenine dinucleotide
NGF	Nerve growth factor
NT-3	Neurotrophin 3
ORR	Optical redox ratio
OXPHOS	Oxidative phosphorylation
TCA	Tricarboxylic acid
TME	Tumor microenvironment
G3P	Glyceraldehyde-3-phosphate
